# Human Neonatal Cardiovascular Progenitors: Unlocking the Secret to Regenerative Ability

**DOI:** 10.1371/journal.pone.0077464

**Published:** 2013-10-28

**Authors:** Tania I. Fuentes, Nancy Appleby, Eric Tsay, J. Julian Martinez, Leonard Bailey, Nahidh Hasaniya, Mary Kearns-Jonker

**Affiliations:** 1 Department of Pathology and Human Anatomy, Loma Linda University School of Medicine, Loma Linda, California, United States of America; 2 Department of Cardiothoracic Surgery, Loma Linda University School of Medicine, Loma Linda, California, United States of America; Centro Cardiologico Monzino, Italy

## Abstract

Although clinical benefit can be achieved after cardiac transplantation of adult c-kit+ or cardiosphere-derived cells for myocardial repair, these stem cells lack the regenerative capacity unique to neonatal cardiovascular stem cells. Unraveling the molecular basis for this age-related discrepancy in function could potentially transform cardiovascular stem cell transplantation. In this report, clonal populations of human neonatal and adult cardiovascular progenitor cells were isolated and characterized, revealing the existence of a novel subpopulation of endogenous cardiovascular stem cells that persist throughout life and co-express both c-kit and isl1. Epigenetic profiling identified 41 microRNAs whose expression was significantly altered with age in phenotypically-matched clones. These differences were correlated with reduced proliferation and a limited capacity to invade in response to growth factor stimulation, despite high levels of growth factor receptor on progenitors isolated from adults. Further understanding of these differences may provide novel therapeutic targets to enhance cardiovascular regenerative capacity.

## Introduction

Endogenous cardiac progenitor cells (CPCs) are being carefully investigated to determine whether they have the ability to repair the heart when expanded *in vitro* and re-administered as a cell-based treatment after myocardial infarction in human clinical trials [1], [2]. As CPCs age, however, they lose the ability to efficiently regenerate damaged heart tissue. Telomerase activity is reduced with chronological age and an associated decline in the number of functionally-competent cardiac progenitor cells results in a dramatic loss of growth reserve within the adult heart [3], [4]. Functional studies in mice have shown that neonatal, not adult, c-kit+ cardiac progenitors support post-infarct myogenesis [5]. The molecular basis underlying the enhanced capacity for regeneration that distinguishes human neonatal cardiovascular progenitor cells from adults has not been defined.

As a fetus matures into a neonate, several developmental changes impact the CPC. Lineage tracing studies using embryonic stem cells show that early cardiovascular progenitors expressing MESP1 differentiate into two separate classes of Nkx2.5+ progenitor populations, one characterized by the expression of Isl1 and another characterized by the absence of Isl1 [6]. The Isl1+ cardiac progenitors can be differentiated into all three cardiac lineages including endothelial cells, smooth muscle cells, and cardiomyocytes [7]. The differentiation capacity of Isl1- CPCs is limited to smooth muscle cells and cardiomyocytes [6]. Histological analysis suggests that cells positive for Isl1, and SSEA-4 (an early stem cell marker) are abundant in the fetus and are only sporadically found in the neonate. Cells expressing c-kit and Nkx2.5 decline in number significantly as a neonate transitions into an infant [8], [9]. A gradual reduction of proliferation occurs in the heart at this time; during the neonatal period there are 3 times as many proliferating cells as those identified in children >2 years of age [9]. After the first month of life, the dynamics of the CPC population changes dramatically, highlighting the neonatal window as an optimal time during which progenitor cells can be isolated for therapy. The biological features that distinguish neonatal cardiovascular progenitor cells in humans will provide new insight that can be used to improve the outcome of stem cell-based treatment.

In this report, the epigenetic, phenotypic and functional changes that distinguish neonatal from adult cardiovascular progenitor cells are detailed within a newly-defined population of Isl1, c-kit co-expressing cardiovascular progenitor cells. By comparing matched, clonal cardiovascular progenitor cell populations that differ only by age, we identify significant differences in microRNA regulation and gene expression that correlate with functional limitations in the adult cardiovascular progenitor cell population.

## Results

### Phenotypic Profiling and Identification of Cardiovascular Progenitor Cell Clones Isolated from Human Neonates and Adults

**Figure 1:**
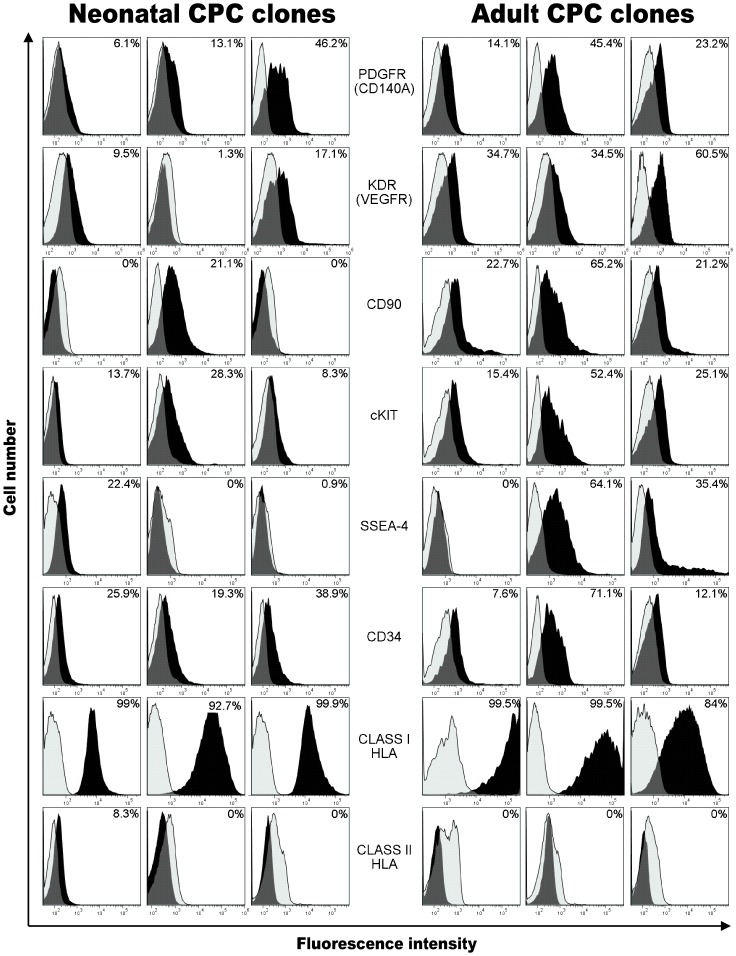
Phenotypic characterization of surface markers on neonatal and adult CPC clones. CPC clones from human neonates and adults were compared using flow cytometry to identify the surface phenotype of these cells. Labeling for select markers on representative CPC clones is shown in the figure where positive staining above the isotype control is shown in black. Each column represents a separate and representative clonal population. The surface phenotype of 24 clones was examined in total.

The surface marker profile of cardiovascular progenitor cell clones residing within the heart of human neonates ≤1 month old and 57–75 year old adults was directly compared by flow cytometry (Figure 1, Table S1). Over 240 cardiovascular cell clones were isolated by single cell expansion. Phenotypic profiling using seventeen different antibodies (Table S2), specific for surface antigens reported to be present on functionally competent cardiovascular progenitors, provided a basis for identifying comparable cardiovascular progenitor cells residing in the heart of both newborns and adults. All clones expressed moderate to high levels of CD105 (60.6–99.8%), CD73 (41.0–98.3%), CD44 (60.6–99.8%), CD13 (73.7–99.9%), IGF1R (58.0–99.1%), and CD146 (35.7–99.9%). c-kit was expressed at lower levels (2.5–52.4%), and expression of KDR (0–75.1%), PDGFR (2.4–57.9%), CD34 (4.9–78.8%) and SSEA4 (0–95.7%) was variable, thereby distinguishing specific populations. Interestingly, the majority of surface antigens were not expressed at significantly different levels in adult and neonatal cardiac progenitors. Of the 17 surface antigens profiled, only CD31 was expressed at significantly lower levels in adult cardiovascular progenitors (p = 0.04). The progenitors were positive for the expression of HLA class I antigens (82.6–99.9%) and HLA class II antigens were either not expressed (0–3.9% in 21 CPC clones) or expressed at low to moderate levels (3 CPC clones 8.3%, 28.6%, 34.1%).

### Coexpression of Isl1 and c-kit on Human Neonatal and Adult Cardiac Progenitors

**Figure 2:**
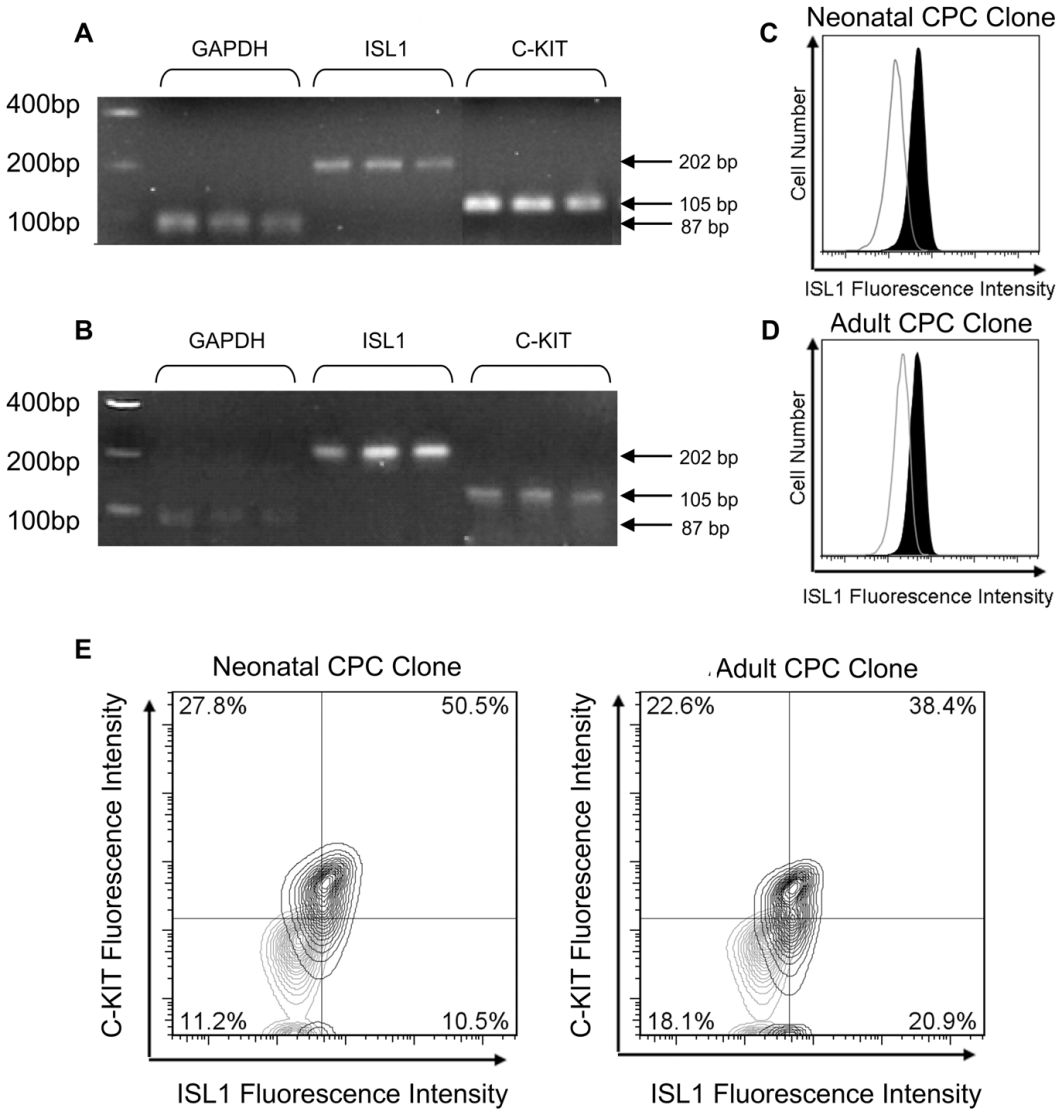
Co-expression of Isl1 and c-kit on neonatal and adult cardiovascular progenitors. Adult and neonatal cardiovascular progenitor cell clones were examined by PCR and flow cytometry to determine whether Isl1 was expressed in these cells. c-kit expression, initially identified by flow cytometry, was confirmed by PCR. PCR products were run on a gel, transcripts for Isl1 (size = 202 bp) and c-kit (size = 105 bp) were co-expressed on both neonatal (A) and adult (B) cardiovascular progenitor cell clones. Flow cytometry confirmed the expression of Isl1 protein (C & D) and the coexpression of Isl1 and c-kit protein (E) on neonatal and adult cardiovascular progenitors.

Expression of Isl1 and c-kit identifies cells with cardiomyogenic potential [10], [11]. Characterization by flow cytometry and PCR revealed that Isl1 and c-kit were co-expressed on CPC clones isolated from both neonates and adults (Figure 2). The majority of CPC clones expressed moderate levels of c-kit (23±3% in neonates, 27±3% in adults). In neonates, Isl1 was present on most, but not all kit+ clones (78%, N = 13). In adults, cardiac progenitor clones were all c-kit+ and Isl1+ (N = 16).

The Isl1+ c-kit+ neonatal and adult CPC clones were differentiated into cardiomyocytes using previously reported protocols [12]. Successful differentiation was supported by the expression of mRNA transcripts for NK2 homeobox 5, gata binding protein 4, cardiac myosin light chain 2, cardiac myosin heavy chain alpha and troponin T, which were induced during the differentiation protocol (N = 12, Figure S1). There were no significant differences in transcription of these proteins between neonatal and adult Isl1+ c-kit+ cardiovascular progenitors. Cardiac progenitor cells were shown by immunocytochemistry to express cardiac Troponin I (Figure S2). When treated with 10 nM dexamethasone for 6 days [13], CPCs were successfully differentiated into all three cardiovascular lineages as demonstrated by a shift in mean fluorescence intensity when the cells were treated with antibodies to identify binding to smooth muscle actin, von Willebrand Factor, cardiac Troponin T and cardiac Troponin I using flow cytometry.

### MicroRNA Profiling Predicts Functional Differences in Neonatal and Adult Cardiovascular Progenitors

**Figure 3:**
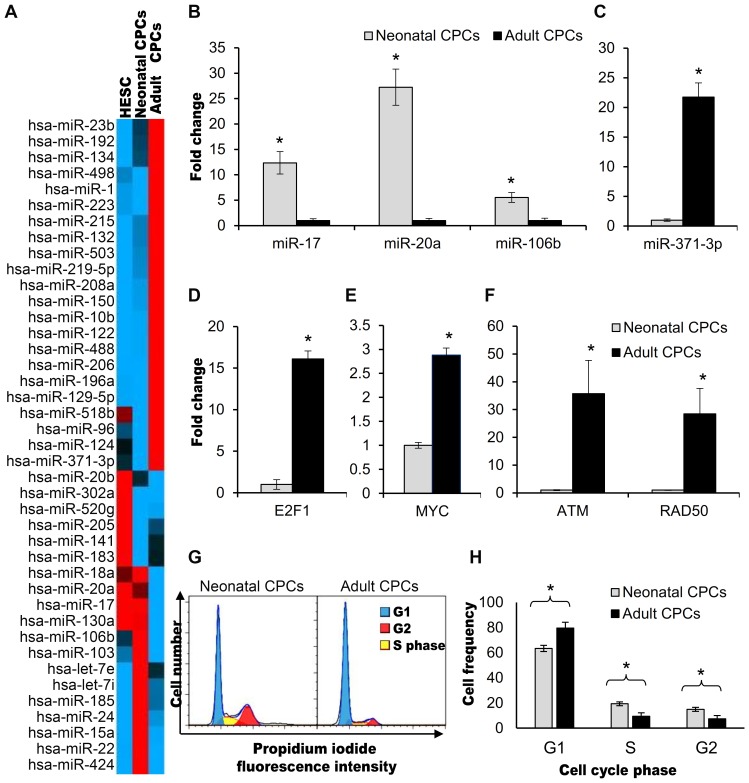
MicroRNA profiling and gene expression predicts functional differences when comparing neonatal and adult cardiovascular progenitors. A) Heat map of 41 microRNAs differentially regulated in neonatal (N = 8) and adult (N = 3) cardiac progenitors. hESC were also profiled as a means for comparison. Red color identifies maximum expression, black color represents average expression, blue color identifies microRNAs with minimum expression. Sets of co-regulated microRNAs were grouped together by RT^2^ Profiler PCR Array Data Analysis Version 3.5 software (SABiosciences). B) MicroRNAs expressed at significantly different levels in adult vs. neonatal CPCs suggest that proliferation is higher in neonatal CPCs. MicroRNA 17, miR-20a, and miR-106 b levels, positively correlated with proliferation, were significantly higher in neonatal CPCs (p = 0.0094, p = 0.0030, p = 0.0085, respectively). C) MicroRNA-371-3p, associated with senescence, was expressed at significantly higher levels in adult CPCs (p = 0.0238). D) Messenger RNA transcripts for E2F1 (N = 6) and E) Myc (N = 5) were significantly higher in adult cardiovascular progenitors (p = 0.0075, p = 0.0086 respectively). E2F1 regulates the G1 to S transition of the cell cycle. F) Expression of DNA repair proteins ATM (N = 5) and RAD50 (N = 5) were significantly higher in adult CPCs (P = 0.0099, P<0.0001, respectively). G) Representative cell cycle analysis of neonatal and adult CPCs. H) Quantification of cell cycle analysis. The frequency of adult CPCs (N = 5) in G1 was significantly higher than neonatal CPCs (P = 0.0038). The frequency of neonatal CPCs (N = 5) in the S and G2 phases of the cell cycle was significantly higher than adult CPCs (p = 0.0066, p = 0.0051 respectively).

Unique differences in epigenetic regulation emerged when comparing cardiovascular progenitor cell clones by microRNA profiling. MicroRNAs (miRNAs) function to negatively regulate mRNA expression by either translational inhibition or degradation. When comparing neonatal and adult CPC clones, 41 out of 88 microRNAs analyzed were expressed at significantly (P<0.05) different levels (Figure 3a, Table S3). MicroRNA expression levels in hES-3 embryonic stem cells were also identified and the results are shown in Figure 3 for comparison. The microRNA expression pattern of neonatal cardiac progenitors was more similar to that of human embryonic stem cells, highlighting a number of shared characteristics.

DIANA mirPath computational software identified forty-six pathways that were significantly (P<0.05) impacted by differentially-expressed microRNAs (Table S4). Fourteen of these pathways were relevant to proliferation, including Wnt signaling, MAPK signaling, p53 signaling, TGF-β signaling, VEGF signaling, and base excision repair. Functional differences in proliferation would be anticipated based on the expression of microRNAs 17, miR-20a, and miR-106b which were expressed at significantly higher levels in neonatal cardiovascular progenitors (Figure 3b) [14]–[17]. High level expression of these microRNAs promotes cell cycle progression by suppressing the inappropriate accumulation of E2F1 transcription factors that lead to G1 arrest [16], [17]. E2F1 regulates the G1 to S transition of the cell cycle to induce proliferation [18]. The 16-fold reduction in mRNA transcripts for E2F1 in neonatal cardiac progenitors was confirmed by RT-PCR (p = 0.0075, Figure 3c).

### Replicative Senescence in Adult Cardiac Progenitors Predicted by microRNA and Gene Expression Differences

Replicative senescence is defined by a progressive loss in proliferative ability despite normal viability and metabolic activity [19] and is associated with an increased DNA damage response and increased cell size [20]. Several microRNAs that were highly expressed in neonatal CPCs are linked to proliferative ability and play a role in preventing cellular senescence. MicroRNAs 20a and 17, which were upregulated in neonatal CPCs (27.3 fold, P = 0.0030 and 12.5 fold, P = 0.0094 Figure 3b), function to rescue cells from Ras-induced cellular senescence [21] and reduce DNA double-stranded breaks [17]. In contrast, microRNA-371-3p, upregulated 27.9 fold (p = 0.0238) in adult cardiovascular progenitor cells, is induced during replicative senescence [22]. Myc expression has been associated with the induction of cellular senescence [23] due to DNA stress [24]. Transcripts for Myc and DNA repair proteins, RAD50 and ATM were significantly elevated in adult cardiac progenitors (2.9 fold, P = 0.0086, 28.5 fold, P<0.0001, 35.7 fold, P = 0.0099 respectively, Figure 3c&d). Comparison of cell size by flow cytometry in three separate experiments using forward scatter gating demonstrated that adult cardiac progenitors had a greater percentage of large cells when compared with neonatal CPCs (56.3% vs. 40.2%, N = 5, p = 0.0073).

### Rate of Progression Through the Cell Cycle Differs in Neonatal and Adult Cardiovascular Progenitors

To investigate the predicted proliferative differences demonstrated by microRNA profiling, propidium iodide (PI), a DNA intercalating agent, was used to identify the percentage of cells in each phase of the cell cycle. Using flow cytometry to detect PI fluorescence intensity, a higher frequency of adult cardiac clones were identified in G1 (82.5% vs. 63.3%, P = 0.0046) and a higher frequency of neonatal clones were identified in S phase (19.4% vs. 7.0%, P = 0.0026) and in G2 (15.0% vs. 7.5%, P = 0.0051) (Figure 3e). Neonatal CPCs proliferate more actively when compared to adult CPCs.

### Cardiovascular Progenitors from Neonates and Adults Differ in their Ability to Respond to Growth Factor Stimulation

**Figure 4:**
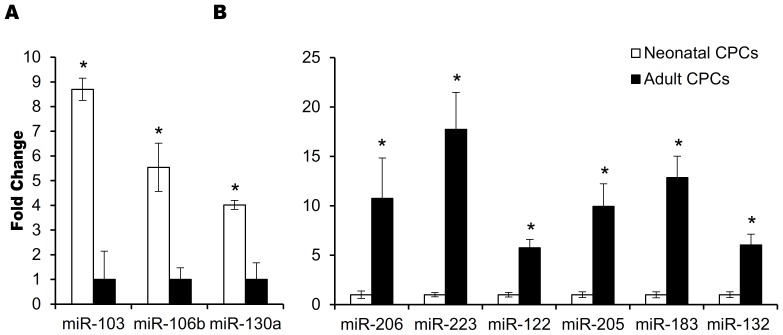
MicroRNAs expressed at significantly different levels (p<0.05) impact invasion. MicroRNAs that both promote (A) and inhibit (B) invasion were expressed at significantly different levels when comparing neonatal and adult CPCs by RT-PCR. MicroRNAs that promote the ability to invade are transcribed at significantly higher levels in neonatal CPCs.

**Figure 5:**
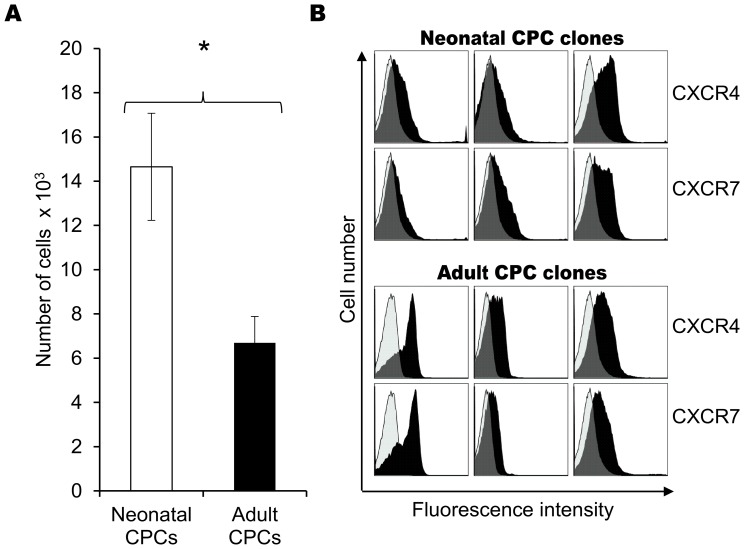
Cell invasion in response to SDF-1. A) Phenotypically similar neonatal and adult clones were run on a transwell invasion assay. The adult CPC clones were less invasive than their neonatal counterparts (n = 5, run in triplicate) in response to SDF-1α, results were significant to P = 0.0463. B) SDF-1α receptors, CXCR4 and CXCR7, were expressed on both neonatal and adult cardiovascular progenitor cell clones. Each column represents a separate and representative clonal population. Isotype control labeling is shown in white, positive labeling is shown in black.

Cardiac regeneration requires CPC migration away from its stem cell niche, followed by invasion into the area of injury in response to external stimuli. Nine microRNAs reported to regulate invasion [25]–[28] were expressed at significantly (P<0.05) different levels in neonatal and adult CPCs (Figure 4). To investigate the possibility that neonatal and adult progenitors differ in their ability to invade the site of injury within the heart, transwell invasion assays were performed to test the response of cells to SDF-1α (stromal cell-derived factor-1). SDF-1α is secreted in the infarcted heart and recruits endogenous cardiac stem cells to the site of injury [29], [30]. Fewer adult cardiac progenitors (6.7×10^3^) were able to invade through the basement membrane extract when compared to neonatal CPCs (14.6×10^3^, p = 0.0463, Figure 5a). The inability of adult CPCs to invade in response to SDF-1α was not due to the lack of SDF-1α receptor expression on the surface of these CPCs, as demonstrated by flow cytometry. The surface expression of CXCR4 and CXCR7, both of which are receptors for SDF-1α [31], was comparable on progenitors isolated from neonates and adults (Figure 5b).

### SSEA-4+ Cardiovascular Progenitors Invade More Readily

**Figure 6:**
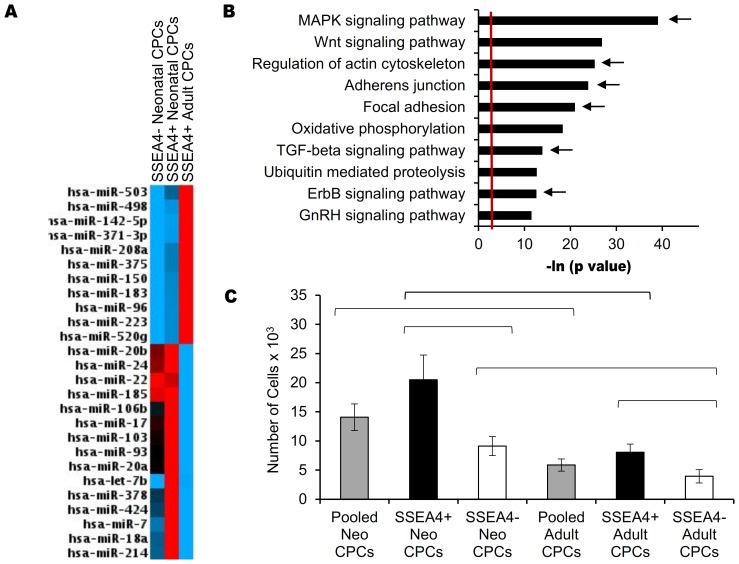
SSEA-4 separates cardiovascular progenitors by the ability to invade. A) Heat map of 26 microRNAs whose expression was differentially regulated in SSEA4+ neonatal and adult cardiovascular progenitors (p<0.05). Red color identifies microRNAs with maximum expression, black color indicates average expression, blue color shows microRNAs with minimum expression. Sets of similarly expressed microRNAs are grouped together by RT^2^ Profiler PCR Array Data Analysis Version 3.5 software (SABiosciences). B) MicroRNAs expressed at significantly different levels when comparing SSEA4+ adult and neonatal CPCs were analyzed using DIANA mirPATH software. The top ten pathways that impact cardiovascular stem cell function, and are regulated by these microRNAs, are shown. Six of the top ten pathways impact invasion and are marked with an arrow. The red line denotes significance of P = 0.05. C) The invasive response of SSEA4+ neonatal and adult cardiovascular progenitor cell clones, run on a transwell migration assay, was compared. Both SSEA4+ neonatal and adult CPCs were significantly more responsive to SDF-1α when compared to SSEA4- neonatal and adult CPC, respectively (P = 0.0186, P = 0.0297). SSEA4+ neonatal cardiovascular progenitor cell clones were most responsive to SDF-1. These cells were significantly more invasive when compared to both SSEA4+ adult and SSEA4- neonatal CPC clones (p = 0.0183, p = 0.0186).

SSEA-4 was not expressed on all CPC clones. Inherent differences were noted in neonatal and adult cardiac progenitors that were found, by flow cytometry, to express SSEA-4. MicroRNA profiling revealed 26 microRNAs expressed at significantly different levels when comparing SSEA4+ adult and neonatal progenitors (P<0.05, Figure 6A, Table S5). The top ten pathways regulated by these microRNAs, and impacting cardiovascular stem cell function, are shown in figure 6b. Six of these pathways impact invasion (marked with an arrow). In functional studies, the SSEA-4+ progenitors were more highly invasive than SSEA4- clones within the neonatal cardiovascular progenitor cell population (2.0×10^4^ vs 9.1×10^3^, p = 0.0186, Figure 6C). Adult CPC clones expressing SSEA4+ were similarly more responsive to SDF-1 (8.1×10^3^ vs 3.9×10^3^, p = 0.0297). SSEA4+ cardiovascular progenitors display an enhanced capacity to invade infarcted tissue in response to injury.

## Discussion

In the present study, Isl1+ c-kit+ cardiovascular progenitor cells, identified in both human neonates and adults, were expanded as clonal populations and utilized as a resource to unravel the phenotypic and epigenetic features that distinguish neonatal and adult cardiac progenitors.

This population of cells was not previously described within the endogenous cardiovascular progenitor cell population in adult humans and challenges current dogma suggesting that Isl1+ CPCs are abundant only in the fetal or neonatal heart [32], [33]. Previous reports comparing neonatal and adult cardiosphere-derived cells (CDCs) for Isl1 expression indicated that Isl1 is abundant in neonatal CDCs but not in CPCs isolated from adults (36.2% of neonatal CPCs versus 3.2% adult CPCs) [32]. Cardiac progenitors co-expressing c-kit and Isl1, identified in the fetal heart [34], have not been isolated from the adult myocardium. Stem cells isolated from the adult heart were reported to have no overlap in expression of c-kit and Isl1 (0.01% coexpression by flow cytometry) [35]. The very existence of an Isl1+ c-kit+ progenitor in human adults has been questioned [36]. Our results demonstrate, for the first time, that the Isl1+ c-kit+ cardiac progenitor phenotype exists in both the neonatal and adult human heart. As in the embryonic heart [34], all progenitors that expressed Isl1 expressed c-kit, however not all c-kit+ cells expressed Isl1, suggesting that the Isl1+ c-kit+ progenitor may be a subpopulation of c-kit+ progenitors. PDGFR and IGF1R, which are present on subpopulations of c-kit+ progenitors with superior regenerative capacity [37], [38], were expressed at moderate to high levels on these cells.

HLA Class II antigens were not expressed on the majority of neonatal and adult CPC clones. MHC class II expression activates acute T-cell mediated graft rejection [39]. Allogeneic transplantation of class I positive, class II negative cardiac progenitors would be expected to elicit a minor, but transient local immune response [40].

Based on findings reported here, the capacity for cardiac regeneration after transplantation of CPCs in neonates and adults is impacted by underlying differences in epigenetic regulation. Forty-one microRNAs were expressed at significantly different levels as a consequence of age. Pathways significantly impacted by these microRNAs fell into broad categories such as proliferation (ex. p53 signaling, base excision repair, MAPK signaling) and migration/invasion (ex. Regulation of Actin Cytoskeleton, TGF-β signaling, VEGF signaling) suggesting that regulatory mechanisms governing these processes differed significantly in neonatal and adult progenitors. In rodents, microRNA profiling similarly identified mechanisms by which a proliferative difference occurs when comparing neonatal and adult CPCs [41]. Human embryonic CPCs directly isolated from the heart without culture have a proliferative advantage over adult CPCs [41]. Our studies take this work a step further by using matched clonal populations to document distinct differences in microRNA expression when comparing neonatal and adult human cardiovascular progenitor cells.

A possible mechanism for decreased proliferation in adult cardiac progenitors is complex regulation of the Myc-E2F1 axis by microRNAs leading to an increase in cellular senescence. E2F1 is a transcriptional activator that is important in the G1/S transition; inappropriate accumulation of E2F1 increases DNA damage response and significantly impacts the ability of cells to enter the S phase of the cell cycle [17]. High levels of Myc expression may result in senescence by initiating cellular stress [23]. This stress leads to upregulation of DNA repair proteins and cell cycle arrest [24], both of which were observed in adult cardiac progenitors. microRNA-371-3p, which was highly expressed in adult CPCs, is correlated with induction of senescence [22]. Pro-proliferative microRNAs such as miR-106b, mir-20a, and mir-17 also play a role; these microRNAs help reduce G1 arrest through regulation of E2F1 transcription factors [14], [17]. Transcripts for all three microRNAs were significantly elevated in neonatal CPCs and mir-17 and mir-20a are directly regulated by Myc expression [17], [42].

MicroRNA profiling and pathway analysis also predicted differences in the capacity to invade, results that were confirmed *in vitro*. Fewer adult CPCs responded to SDF-1α, despite having adequate CXCR4 and CXCR7 receptor levels on their surface. SDF-1α is secreted in the damaged heart and recruits both exogenous and endogenous cardiovascular stem cells to the site of injury [30]. Interestingly, not all neonatal and adult cardiac progenitors responded equally to SDF-1α; we identified a subpopulation of neonatal and adult progenitors that expressed SSEA-4. SSEA-4 is a stem cell marker that identifies cells in early stages of progenitor development [43]. In our study, SSEA-4 expression was correlated with differential expression of microRNAs involved in invasion-related pathways. SSEA-4 is expressed on cancer cells that are more highly invasive [44], however little is known about the impact of SSEA-4 on SDF-1α-induced invasion in cardiovascular cells. Our study shows that SSEA-4+ progenitors invaded more readily in response to SDF-1α when compared to their SSEA-4- counterparts. Comparison of SSEA4+ neonatal and adult CPCs demonstrated that age negatively impacts invasion.

There may be several reasons for this age-related functional discrepancy. SDF-1α signals primarily through Akt signaling or ERK1/2 signaling. Depending on the cell type, these pathways can signal independently, one regulating the survival and proliferation functions of SDF-1 signaling, the other regulating invasion and migration [45]. After activation by SDF-1α, CXCR4 dimerizes and is phosphorylated by JAK2 and JAK3 which create docking sites for transcription factors to propagate signaling [46]. Adult CPCs may demonstrate decreased CXCR4 dimerization, reduced numbers of cosignaling molecules, or lower levels of phosphorylation and activation of the receptor. Additionally, the cells themselves may secrete different levels of growth factors which could contribute to lower levels of receptor activation.

MicroRNAs play a role in SDF-1α signaling and invasion. Significant differences in microRNA expression in aged cardiac progenitors influences functional parameters relevant for cardiovascular repair. For example, activation of the SDF-1α receptor, CXCR4, induces the expression of proteases such as matrix metallopeptidase 9 (MMP9) which help to degrade the extracellular matrix, allowing cells to invade [47]. The expression of MMP9 is inhibited by miR-132 [26] and miR-206 [48]. The expression of these microRNAs were significantly upregulated in adult CPCs when compared with their expression level in neonatal CPCs (6.0 fold, p = 0.0005, 9.6 fold, p = 0.0166 respectively). Inhibitors of invasion, such as metallopeptidase inhibitor (TIMP3) [27] and homeo box A5 (HOXA5) [49] are targeted by microRNAs upregulated in neonatal cardiac progenitors. microRNA-103 represses the expression of TIMP-3 [27] (elevated 8.7 fold in neonatal CPCs, p = 0.0027) and microRNA-130a represses the expression of HOXA5 [50] (elevated 2.4 fold in neonatal CPCs, p = 0.0288). If adult progenitors cannot effectively invade the site of injury, a dramatic difference in regeneration will occur with age.

MicroRNA-mediated regulation of gene expression is a novel, rapidly expanding area of research which has opened up new therapeutic options for the reversal of heart disease. Pretreatment of cardiac progenitors prior to transplantation, or direct administration of microRNA therapeutics into the heart may activate stem cell recruitment from endogenous sources. In neonatal rodents, microRNA mimics had a positive effect on cytokinesis, DNA synthesis, and cell cycle re-entry [51]. MicroRNAs whose expression levels are altered in aged CPCs may be manipulated *in vivo* to promote recovery from myocardial damage. For example mir-24 (upregulated 8.4 fold in neonatal CPCs) when introduced after myocardial infarction reduces infarct size [52]. Conversely, inhibition of mir-208a (upregulated 12.4 fold in adult CPCs) reduced cardiac remodeling, improved cardiac function, and survival after hypertension-induced heart failure [53]. The potential for microRNA-based therapeutics to promote stem cell mobilization, combined with an understanding of the role of microRNAs in cardiac regeneration, promises to open up new treatment options that may improve the outcome of stem cell-based therapies.

Cardiovascular progenitor cells co-expressing c-kit and Isl1 can be identified and expanded *in vitro* from neonatal and adult heart tissue. Epigenetic differences highlight the mechanism by which neonatal cardiovascular progenitor cells can proliferate and invade in response to cytokine stimulation, whereas the adult cells have a diminished capacity for mobilization. Neonatal cardiac progenitor cell clones expressing SSEA-4 are more responsive to stimulation and may be optimal for cardiac regeneration.

## Methods

### Ethics Statement/Cell Isolation and Expansion

The Institutional Review Board of Loma Linda University approved the protocol for use of tissue that was discarded during cardiovascular surgery, without identifiable private information, for this study with a waiver of informed consent. Discarded atrial cardiac tissue from human neonates (<1 month old) and adults (57–75 years old), was cut into small clumps (approximately 1 mm^3^) and collagenase digested (Roche Applied Science, Indianapolis, IN) for approximately 2 hours at 37 degrees at a proportion of 1∶2.5 tissue volume vs. collagenase. This solution was then passed through a 40 µm cell strainer to isolate cardiac progenitors [12]. Resulting cells were cloned by limiting dilution at a concentration of 0.8 cells per well to create clonal populations which were expanded for further study. Over two hundred and forty clones were isolated from human patients by this procedure; seventeen neonatal and sixteen adult cardiovascular cell clones were compared in detail for this study. The human embryonic stem cell line hES-3 was cultured as previously published [54]. Cardiac progenitor cell clones were differentiated both by treatment with 5-azacytidine followed by ascorbic acid and TGF-β to induce cardiomyogenic differentiation [12] and by treatment with 10 nM dexamethasone in DMEM/F12 media supplemented with 10% fetal bovine serum to induce differentiation into all three cardiovascular lineages [13]. Cardiomyogenic differentiation was confirmed by measuring the induction of mature cardiac-specific transcripts by RT-PCR. Expression of endothelial, smooth muscle, and cardiomyocyte markers induced by dexamethasone treatment were quantified by flow cytometry.

### Flow Cytometry Experiments

Cells were labeled using antibody concentrations that were recommended by the manufacturer(s). Fluorescently labeled cells were analyzed using a MACSquant analyzer (Miltenyi Biotec, Auburn, CA). FlowJo software (Ashland, OR) was used for quantification. Dead cells and small particles were gated out using forward-scatter, side-scatter gating. Isotype controls (MS IgG1) were used to define negative and positive populations. Antibodies used included CD105-PE, IGF1R-PE, CXCR4-PE, CXCR7-PE, CD140a-PE, CD146-PE, SSEA-4-FITC, Pan HLA-FITC, CD309-PerCP/Cy5.5 (Biolegend, San Diego, CA), CD44-FITC, CD13-PE, CD31-PE, HLA-Dr-PE, CD73-PE, CD34-PE (BD Biosciences San Jose, CA), CD90-PE (Immunotech, Brea, CA), CD117-PE (Millipore, Billerica, MA). Additional antibody data can be found in Table S2. Relative percentage of large neonatal and adult cardiovascular progenitors was assessed by flow cytometry. Large cells were defined by forward scatter gating that was uniformly applied to all samples and the percentage of cells within this gate was compared. Each test was run in duplicate.

### RT-PCR

Total RNA was extracted from neonatal and adult cardiovascular progenitor cell clones and reverse transcribed into cDNA using superscript III (Invitrogen, Grand Island, NY). Real-time PCR was performed using Go Taq® Green Master Mix (Promega, Madison, WI). The PCR conditions were: 94°C for 10 minutes, 94°C for 15 seconds, 56°C for 60 seconds, 72°C for 30 seconds for a total of 40 cycles. Human primers were created using NCBI primer blast and included tyrosine-protein kinase (c-kit), glyceraldehyde-3-phosphate dehydrogenase (GAPDH), and ISL LIM homeobox 1 (Isl1). Primer sequences are listed in Table S6.

For microRNA profiling experiments, total RNA was extracted from representative neonatal (N = 8) and adult (N = 3) CPC clones and converted to cDNA using the RT^2^ miRNA First Strand Kit (SABiosciences, Valencia, CA). The Cell Development & Differentiation miRNA PCR array plates (SABiosciences, Valencia CA) and RT^2^ SYBR Green qPCR Mastermix (SABiosciences, Valencia, CA) was used. Plates were run on an iCycler iQ5 PCR Thermal Cycler (Bio-Rad, Hercules, CA) for 94°C for 10 minutes, 94°C for 15 seconds, 60°C for 60 seconds, 72°C for 30 seconds for a total of 40 cycles. Fold change was calculated using the ΔΔCt method [55]. Data from representative cardiovascular cell clones was analyzed individually, then pooled within age groups. The microRNA expression data has been deposited in NCBI’s Gene Expression Omnibus [56] and are accessible through GEO series accession number GSE49235. miRNAs that were expressed at significantly different levels when comparing neonatal and adult CPCs were analyzed using DIANA mirPath computational software (Athens, Greece) which performs an enrichment analysis of multiple microRNA target genes, comparing each set of microRNA targets to all known KEGG pathways [57].

### Cell Cycle Analysis

Cells were trypsinized at 60–80% confluency, counted, and concentrated to 10^5^ cells/0.3 ml PBS. Ethanol (0.7 ml) was added dropwise to fix the cells. Cells were stored for at least 1 hour at -20 degrees, washed, and incubated at 37 degrees with RNase A (0.5 mg/ml Invitrogen, Grand Island, NY) for one hour. Propidium iodide was then added at a final concentration of 10 µg/ml. Samples were run on MACSquant analyzer (Miltenyi BIotec, Auburn, CA). Cell cycle analysis was done using FlowJo software (Ashland, OR).

### Transwell Invasion Assay

Cells were plated in the top well of Costar Transwell plates (8 µm pores), coated with Cultrex™ basement membrane extract (Trevigen, Gaithersburg, MD) mimicking extracellular matrix. Cells were plated at a density of 100,000 cells per 100 µl in starved medium. Stromal cell-derived factor-1α (SDF-1α, Invitrogen, Grand Island, NY) was used as a chemoattractant at a concentration of 100 ng per ml of M199 plus EGM-2 in the bottom chamber of a transwell plate. After 24 hours, cells in the bottom wells were trypsinized and counted using a flow cytometer. The response of neonatal and adult CPC clones was analyzed individually then pooled by age and subdivided according to the presence or absence of SSEA-4 expression.

### Immunocytochemistry

Neonatal and adult CPCs were plated on gelatin coated Lab-Tek II cc^2^ chamber slides (Nunc, Rochester, NY) and grown at 37° for 4 days, fixed in 4% paraformaldehyde and stained using a primary anti-troponin I antibody at 10 µg/ml (Millipore #MAB1691, Billerica, MA) and FITC-conjugated, goat anti-mouse IgG secondary antibody at 2 µg/ml (Southern Biotech, Birmingham, Alabama). Coverslips were mounted using Prolong Gold antifade with DAPI (Invitrogen, Grand Island, NY). Slides were imaged using a Zeiss confocal LSM 710 NLO laser-scanning, confocal microscope (GmbH, Germany).

### Statistics

RT^2^ Profiler PCR Array Data Analysis software (SABiosciences, version 3.5) was used to calculate statistical significance for microRNA profiling. Data was tested for normal distribution using the Anderson-Darling normality test calculator Version 6.0 with an alpha of 0.05 (faculty.missouri.edu/∼glaserr/3700s11/AD-Test_Calculator.xls). For normally distributed data, an unpaired, two-tailed student’s t test was performed. For data that was not normally distributed, a Mann-Whitney Rank Sum Test was performed. Statistically significant differences were identified as P<0.05. Data was reported as the mean +/− standard error.

## Supporting Information

Figure S1 **Expression of cardiac Troponin T, Nkx2.5, Gata-4, MLC2v and MHCα is induced after differentiation of Isl1+ c-kit+ cardiac progenitor clones.**
(TIF)

Figure S2 **Expression of cardiac Troponin I in Isl1+ c-kit+ cardiac progenitor clones.**
(TIF)

Table S1 **Cell surface phenotype of neonatal and adult CPCs as identified by flow cytometry.**
(PDF)

Table S2 **Antibodies used to characterize cell surface markers expressed on neonatal and adult CPC clones.**
(PDF)

Table S3 **Relative expression of significantly altered microRNAs in neonatal and adult cardiovascular progenitors.**
(PDF)

Table S4 **Pathway analysis associated with microRNAs that were differentially regulated when comparing neonatal and adult CPCs.**
(PDF)

Table S5 **Relative expression of significantly altered microRNAs in SSEA-4+ neonatal and adult cardiovascular progenitors.**
(PDF)

Table S6 **Primers used to detect gene expression by PCR.**
(PDF)
