# Supplementary figures and images for: Human Neonatal Cardiovascular Progenitors: Unlocking the Secret to Regenerative Ability
Source: PLoS One. 2013 Oct 28;8(10):e77464. doi: 10.1371/journal.pone.0077464 (PMC3810469; doi:10.1371/journal.pone.0077464)

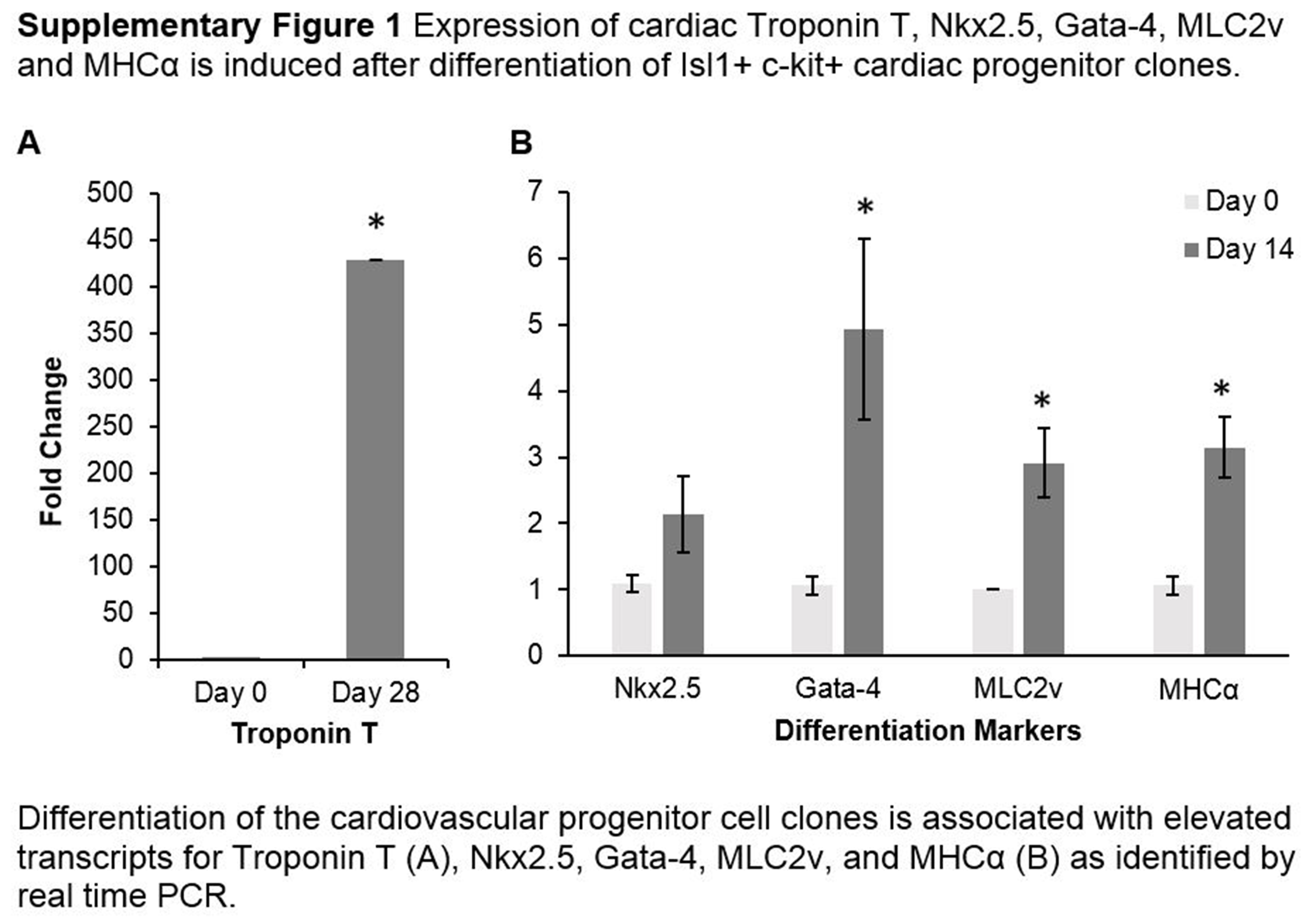

Supplement: Figure S1 — Expression of cardiac Troponin T, Nkx2.5, Gata-4, MLC2v and MHCα is induced after differentiation of Isl1+ c-kit+ cardiac progenitor clones. (TIF) [file pone.0077464.s001.tif]

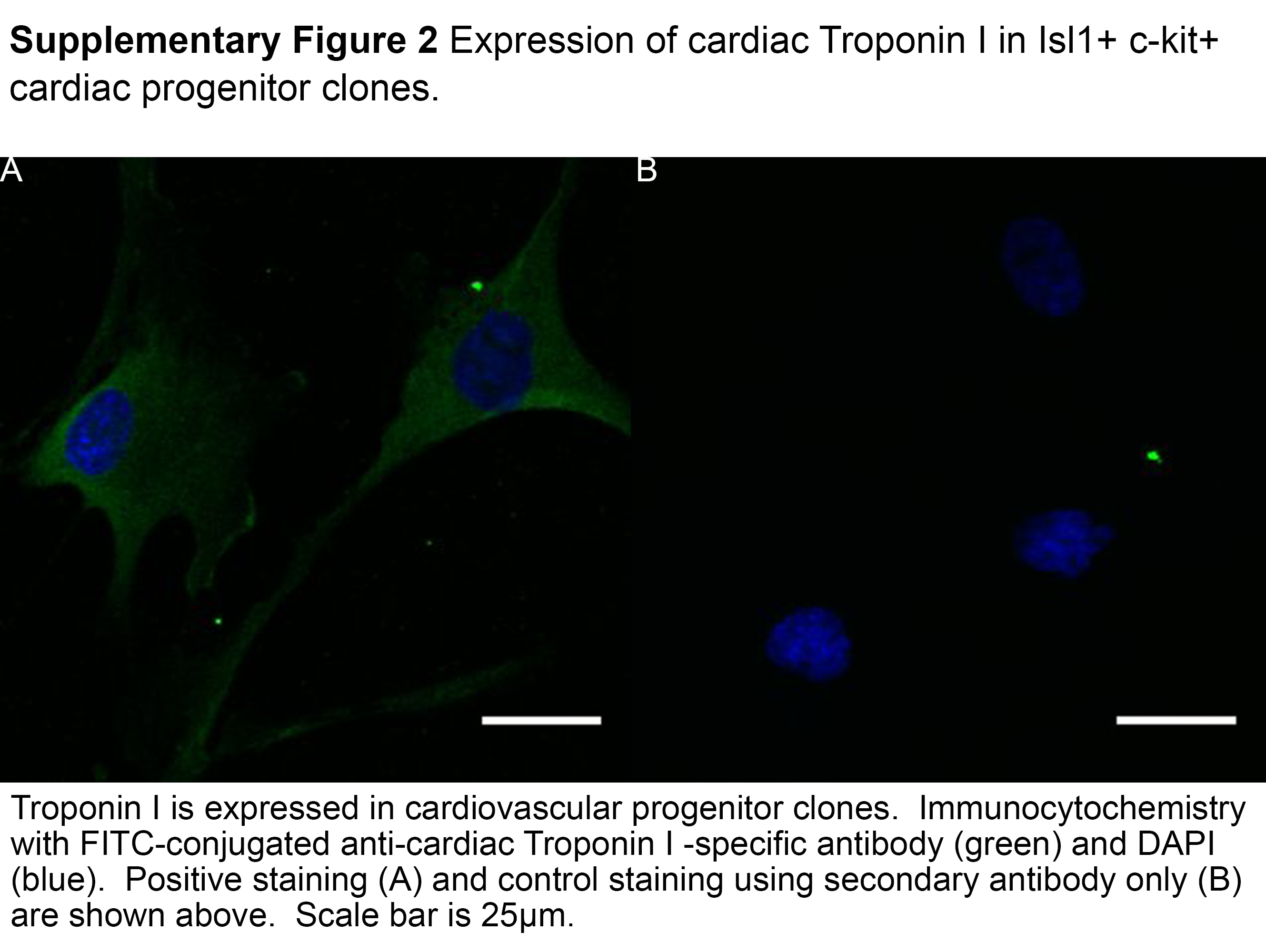

Supplement: Figure S2 — Expression of cardiac Troponin I in Isl1+ c-kit+ cardiac progenitor clones. (TIF) [file pone.0077464.s002.tif]
