# Supplementary material for: Human Neonatal Cardiovascular Progenitors: Unlocking the Secret to Regenerative Ability
Source: PLoS One. 2013 Oct 28;8(10):e77464. doi: 10.1371/journal.pone.0077464 (PMC3810469; doi:10.1371/journal.pone.0077464)
Supplement: Table S1 — Cell surface phenotype of neonatal and adult CPCs as identified by flow cytometry. (PDF) [file pone.0077464.s003.pdf]

Table S1 – Cell surface phenotype of neonatal and adult CPCs as identified by flow cytometry.

Surface Antigen	**Neonatal CPC** Percent labeled	**Adult CPC** Percent labeled	**Range**
CD13	89 ± 2%	98 ± 1%	74 – 100%
CD31	81 ± 8%	43 ± 15%	21 – 95%
CD34	42 ± 5%	25 ± 7%	5 – 79%
CD44	79 ± 6%	89 ± 2%	61 – 100%
CD73	91 ± 3%	88 ± 3%	41 – 98%
CD90	19 ± 6%	32 ± 5%	0 – 95%
CD105	94 ± 2%	92 ± 2%	61 – 99%
CD146	85 ± 4%	96 ± 2%	36 – 100%
C-KIT	23 ± 3%	27 ± 3%	3 – 52.4%
CXCR4	37 ± 6%	50 ± 5%	13 –72%
CXCR7	29 ± 6%	44 ± 7%	3 – 69%
HLA I	87 ± 4%	92 ± 3%	83 – 100 %
HLA II	4 ± 2%	6 ± 4%	0 – 34%
IGF1R	87 ± 3%	89 ± 3%	58 – 99%
KDR	13 ± 7%	35 ± 14%	0 – 75%
PDGFR	20 ± 5%	35 ± 6%	2 – 58%
SSEA-4	12 ± 6%	27 ± 8%	0 – 96%
