# Supplementary material for: Human Neonatal Cardiovascular Progenitors: Unlocking the Secret to Regenerative Ability
Source: PLoS One. 2013 Oct 28;8(10):e77464. doi: 10.1371/journal.pone.0077464 (PMC3810469; doi:10.1371/journal.pone.0077464)
Supplement: Table S2 — Antibodies used to characterize cell surface markers expressed on neonatal and adult CPC clones. (PDF) [file pone.0077464.s004.pdf]

Table S2- Antibodies used to characterize cell surface markers expressed on neonatal and adult CPC clones.

Antibody Against	Fluorophore	Company	Clone	Isotype	Cat #	Lot#
CD44	FITC	BD Biosciences	L178	MS IgG1	347943	502666
CD90	PE	Immunotech	F15-42-1-5	MS IgG1	1840	3
CD13	PE	BD Biosciences	L138	MS IgG1	347837	12616
CD31	PE	BD Biosciences	WM59	Ms IgG1	555446	42184
HLA-Dr	PE	BD Biosciences	G46-6	MS IgG2a	347367	60070
CD73	PE	BD Biosciences	AD2	MS IgG1	550257	77302
CD34	PE	BD Biosciences	8G12	MS IgG1	348057	60098
IGF1R	PE	Biolegend	1H7/CD221	MS IgG1	351805	8152252
CD105	PE	Biolegend	43A3	MS IgG1	323205	B152252
CXCR7	PE	Biolegend	8F11-M16	MS IgG2b	331103	B146742
CD140a	PE	Biolegend	16A1	MS IgG1	323505	B154801
CD146	PE	Biolegend	SHM-57	MS IgG2a	342003	B140547
CXCR4	PE	Biolegend	12G5	MS IgG2a	306505	B139046
SSEA4	FITC	Biolegend	MC-813-70	MS IgG3	330409	B153640
Pan HLA	FITC	Biolegend	W6/32	MS IgG2a	311403	B150905
CD117	PE	Millipore	104D2	IgG1	FCKMAB214P	1995601
CD309	PerCP/Cy5.5	Biolegend	HKDR-1	MS IgG1	338915	B155679
IgG1	PE	BD Biosciences	G18-145	MS IgG1	555787	0000028013
IgG1	FITC	Sigma-Aldrich	MOPC 21	MS IgG1	F6397	015K4830
IgG1	PerCP	Biolegend	MOPC 21	MS IgG1	40018	B137528
ISL1	Mouse Monoclonal	Abcam	1H9	MS IgG1	Ab86472	GR120150-1
vWF	Rabbit Polyclonal	Dako	Polyclonal	n/a	A0082	00088175
SMA	Mouse Monoclonal	Dako	1A4	MS IgG2a kappa	M0851	00087780
Goat anti mouse IgG	FITC	Southern Biotech	Polyclonal	IgG	1030-02	D240-U141C
Goat anti Rabbit Ig	FITC	BD Biosciences	Polyclonal	Ig	554020	2200705
