# Supplementary material for: Human Neonatal Cardiovascular Progenitors: Unlocking the Secret to Regenerative Ability
Source: PLoS One. 2013 Oct 28;8(10):e77464. doi: 10.1371/journal.pone.0077464 (PMC3810469; doi:10.1371/journal.pone.0077464)
Supplement: Table S3 — Relative expression of significantly altered microRNAs in neonatal and adult cardiovascular progenitors. (PDF) [file pone.0077464.s005.pdf]

Table S3 – Relative expression of significantly altered microRNAs in neonatal and adult cardiovascular progenitors.

	Fold Change		St Error		
MicroRNA	Adult CPCs	Neonatal CPCs	Adult CPCs	Neonatal CPCs	P value
miR-22	1	26.87	0.01	0.16	0.0052
miR-424	1	23.92	0.02	0.25	0.0357
miR-20a	1	20.03	0.02	0.18	0.0084
miR-18a	1	14.74	0.03	0.44	0.0238
miR-20b	1	13.01	0.02	0.24	0.0351
miR-17	1	12.45	0.03	0.18	0.0094
miR-15a	1	9.48	0.08	0.23	0.0368
miR-103	1	8.70	0.05	0.13	0.0027
miR-24	1	8.41	0.05	0.10	0.0005
let-7i	1	6.53	0.03	0.37	0.0238
miR-106b	1	4.98	0.09	0.20	0.0295
miR-185	1	3.52	0.06	0.10	0.0022
miR-130a	1	2.41	0.28	0.08	0.0288
let-7e	1	2.23	0.17	0.14	0.0456
miR-192	2.16	1	0.11	0.28	0.0273
miR-134	3.13	1	0.44	0.16	0.0007
miR-23b	3.33	1	1.29	0.25	0.0395
miR-503	4.97	1	0.89	0.45	0.0238
miR-122	4.98	1	0.85	0.24	0.0005
miR-10b	5.48	1	2.34	0.36	0.0279
miR-219-5p	5.98	1	1.75	0.27	0.0045
miR-132	6.03	1	1.10	0.29	0.0005
miR-215	6.80	1	2.70	0.22	0.0145
miR-1	6.85	1	2.22	0.50	0.0238
miR-205	7.65	1	2.30	0.29	0.0038
miR-183	8.11	1	2.16	0.31	0.0020
miR-498	8.21	1	1.40	0.62	0.0238
miR-206	9.59	1	4.10	0.38	0.0166
miR-141	9.70	1	4.02	0.27	0.0137
miR-302a	9.98	1	4.20	0.30	0.0145
miR-520g	10.30	1	1.76	0.33	0.0001
miR-96	12.30	1	0.48	0.27	<0.0001
miR-208a	12.43	1	2.60	0.37	0.0004
miR-223	15.90	1	3.75	0.22	0.0005
miR-124	16.49	1	6.32	0.26	0.0074
miR-150	16.54	1	1.97	0.25	<0.0001
miR-488	17.74	1	7.93	0.50	0.0238
miR-371-3p	21.76	1	2.38	0.21	0.0238
miR-129-5p	23.28	1	10.81	0.18	0.0163
miR-196a	24.02	1	8.76	0.39	0.0052
miR-518b	45.95	1	21.44	0.19	0.0150
