# Supplementary material for: Human Neonatal Cardiovascular Progenitors: Unlocking the Secret to Regenerative Ability
Source: PLoS One. 2013 Oct 28;8(10):e77464. doi: 10.1371/journal.pone.0077464 (PMC3810469; doi:10.1371/journal.pone.0077464)
Supplement: Table S4 — Pathway analysis associated with microRNAs that were differentially regulated when comparing neonatal and adult CPCs. (PDF) [file pone.0077464.s006.pdf]

Supplementary Table 4 – Pathway analysis associated with microRNAs that were differentially regulated when comparing neonatal and adult CPCs

Pathway	-ln(pvalue)*
MAPK signaling pathway	26.14
Ribosome	25.29
Regulation of actin cytoskeleton	23.61
Wnt signaling pathway	21.31
Adherens junction	21.23
Focal adhesion	20.5
Oxidative phosphorylation	17.34
TGF-beta signaling pathway	16.81
ErbB signaling pathway	12.21
Tight junction	10.14
Long-term potentiation	10.13
Ubiquitin mediated proteolysis	10.04
Arachidonic acid metabolism	9.77
Insulin signaling pathway	9.47
mTOR signaling pathway	8.59
p53 signaling pathway	8.52
Glycolysis / Gluconeogenesis	7.46
Androgen and estrogen metabolism	7.36
Pyruvate metabolism	7.31
GnRH signaling pathway	7.23
SNARE interactions in vesicular transport	6.99
Circadian rhythm	6.37
Complement and coagulation cascades	6.04

Tryptophan metabolism	5.73
Polyunsaturated fatty acid biosynthesis	5.53
Gap junction	5.38
Neuroactive ligand-receptor interaction	5.35
ECM-receptor interaction	5.33
Glycine, serine and threonine metabolism	5.3
Linoleic acid metabolism	5.14
Proteasome	4.74
Calcium signaling pathway	4.5
VEGF signaling pathway	4.32
Glycan structures – degradation	4.24
Methionine metabolism	4.24
Starch and sucrose metabolism	4.2
Glutathione metabolism	4.08
Phosphatidylinositol signaling system	4.01
Notch signaling pathway	3.88
Base excision repair	3.65
Adipocytokine signaling pathway	3.65
Valine, leucine and isoleucine degradation	3.64
Histidine metabolism	3.39
D-Glutamine and D-glutamate metabolism	3.12
Butanoate metabolism	3.1
Propanoate metabolism	3.04

*Only pathways found to be significantly different ($p<0.05$) are displayed and are listed from most significant to least significant.
