# Supplementary material for: Human Neonatal Cardiovascular Progenitors: Unlocking the Secret to Regenerative Ability
Source: PLoS One. 2013 Oct 28;8(10):e77464. doi: 10.1371/journal.pone.0077464 (PMC3810469; doi:10.1371/journal.pone.0077464)
Supplement: Table S5 — Relative expression of significantly altered microRNAs in SSEA-4+ neonatal and adult cardiovascular progenitors. (PDF) [file pone.0077464.s007.pdf]

Table S5 – Relative expression of significantly altered microRNAs in SSEA-4+ neonatal and adult cardiovascular progenitors

	Fold Change		St Error		
MicroRNA	SSEA-4+ Adult CPCs	SSEA-4+ Neonatal CPCs	SSEA-4+ Adult CPCs	SSEA-4+ Neonatal CPCs	P value
miR-142-5p	18.71	1	4.12	0.18	0.0127
miR-371-3p	13.36	1	1.46	0.20	0.0011
miR-498	8.81	1	1.50	0.21	0.0067
miR-223	7.59	1	1.79	0.27	0.0219
miR-96	7.23	1	0.28	0.03	<0.0001
miR-183	6.81	1	1.81	0.06	0.0327
miR-375	6.21	1	1.04	0.20	0.0079
miR-520g	6.03	1	1.03	0.28	0.0091
miR-150	5.77	1	0.69	0.46	0.0045
miR-208a	5.51	1	1.15	0.28	0.0189
miR-503	3.12	1	0.56	0.42	0.0383
let-7b	1	2.81	0.15	0.01	0.0117
miR-185	1	3.80	0.06	0.19	0.0217
miR-93	1	4.02	0.10	0.20	0.0268
miR-214	1	4.37	0.09	0.14	0.0101
miR-378	1	6.00	0.12	0.21	0.0259
miR-106b	1	7.91	0.06	0.07	0.0006
miR-24	1	10.63	0.04	0.13	0.0027
miR-7	1	12.59	0.04	0.32	0.0476
miR-103	1	14.18	0.03	0.17	0.0062
miR-17	1	16.46	0.02	0.12	0.0013
miR-20b	1	17.50	0.01	0.28	0.0268
miR-22	1	27.81	0.01	0.32	0.0412
miR-20a	1	31.21	0.01	0.04	<0.0001
miR-18a	1	31.89	0.02	0.25	0.0174
miR-424	1	57.54	0.01	0.18	0.0058
