# Supplementary material for: Human Neonatal Cardiovascular Progenitors: Unlocking the Secret to Regenerative Ability
Source: PLoS One. 2013 Oct 28;8(10):e77464. doi: 10.1371/journal.pone.0077464 (PMC3810469; doi:10.1371/journal.pone.0077464)
Supplement: Table S6 — Primers used to detect gene expression by PCR. (PDF) [file pone.0077464.s008.pdf]

Table S6 – Primers used to detect gene expression by PCR

Gene	Forward Primer	Reverse Primer
ATM	GGGCGAGCCGCAAACGCTAA	TTCGGCCCGTCGGAGCAAAC
C-KIT	ATTCCCAAGCCCATGAGTCCTTGA	ACACGTGGAACACCATCCT
E2F1	GACCATCAGTACCTGGCCGAGAG	GACGACACCGTCAGCCGAGTG
GAPDH	TGCACCACCAACTGCTTAGC	GGCATGGACTGTGGTCATGAG
GATA 4	GATCTTCGCGACAGTTCCTC	GTCCCCGGGAAGGAGAAG
ISL1	CACAAGCGTCTCGGGATTGTGTTT	AGTGGCAAGTCTTCCGACAA
MHCα	GTCATTGCTGAAACCGAGAATG	GCAAAGTACTGGATGACACGCT
MLC-2v	TATTGGAACATGGCCTCTGGAT	GGTGCTGAAGGCTGATTACGTT
MYC	AAGACAGCGGCAGCCCGAAC	TGGGCGAGCTGCTGTCGTTG
RAD50	CTACGGCTTTGCGTCCCCGG	ACACCAGCTGCTTTCCCCGC
TROP T	GTGGGAAGAGGCAGACTGAG	ATAGATGCTCTGCCACAGC
